# Reperfusion Strategy of ST-Elevation Myocardial Infarction: A Meta-Analysis of Primary Percutaneous Coronary Intervention and Pharmaco-Invasive Therapy

**DOI:** 10.3389/fcvm.2022.813325

**Published:** 2022-03-17

**Authors:** Kaiyin Li, Bin Zhang, Bo Zheng, Yan Zhang, Yong Huo

**Affiliations:** ^1^Department of Cardiology, Peking University First Hospital, Beijing, China; ^2^Institute of Cardiovascular Disease, Peking University First hospital, Beijing, China

**Keywords:** ST-elevation myocardial infarction (STEMI), primary percutaneous coronary intervention (PPCI), pharmaco-invasive therapy, mortality, hemorrhage, heart failure, stroke

## Abstract

**Background:**

Pharmaco-invasive therapy (PIT), combining thrombolysis and percutaneous coronary intervention, was a potential complement for primary percutaneous coronary intervention (pPCI), while bleeding risk was still a concern.

**Objectives:**

This study aims to compare the efficacy and safety outcomes of PIT and pPCI.

**Methods:**

A systematic search for randomized controlled trials (RCTs) and observational studies were conducted on Pubmed, Embase, Cochrane library, and Scopus. RCTs and observational studies were all collected and respectively analyzed, and combined pooled analysis was also presented. The primary efficacy outcome was short-term all-cause mortality within 30 days, including in-hospital period. The primary safety outcome was 30-day trial-defined major bleeding events.

**Results:**

A total of 26,597 patients from 5 RCTs and 12 observational studies were included. There was no significant difference in short-term mortality [RCTs: risk ratio (*RR*): 1.14, 95% CI: 0.67–1.93, *I*^2^ = 0%, *p* = 0.64; combined results: odds ratio (OR): 1.09, 95% CI: 0.93–1.29, *I*^2^ = 0%, *p* = 0.30] and 30-day major bleeding events (RCTs: RR: 0.44, 95% CI: 0.07–2.93, *I*^2^ = 0%, *p* = 0.39; combined results: OR: 1.01, 95% CI: 0.53–1.92, *I*^2^ = 0%, *p* = 0.98). However, pPCI reduced risk of in-hospital major bleeding events, stroke and intracranial bleeding, but increased risk of in-hospital heart failure and 30-day heart failure in combined analysis of RCTs and observational studies, despite no significant difference in analysis of RCTs.

**Conclusion:**

Pharmaco-invasive therapy could be an important complement for pPCI in real-world clinical practice under specific conditions, but studies aiming at optimizing thrombolysis and its combination of mandatory coronary angiography are also warranted.

## Introduction

Early reperfusion is crucial for the management of ST-elevation myocardial infarction (STEMI). According to current guidelines, primary percutaneous coronary intervention (pPCI) is the preferred strategy for patients within 12 h of symptom onset (1–3). However, a significant delay of pPCI may attenuate the benefit of myocardial reperfusion (4). Pharmaco-invasive therapy (PIT), an alternative strategy for reperfusion in the management of STEMI, is generally initiated in a prehospital setting or at a non-percutaneous coronary intervention (PCI)-capable hospital with intravenous thrombolysis; rescue PCI is recommended in cases of failed fibrinolysis or there is evidence of re-occlusion or reinfarction with recurrence of ST-segment elevation, and routine angiography is also recommended within a time-window of 2–24 h in cases of successful fibrinolysis. In previous studies, PIT strategy shared similar 30-day clinical outcomes and 1-year mortality with pPCI strategy (5, 6), while some researchers argued that the uncertainty of efficacy and potential increase of bleeding risk were still great concerns (7). Considering the relatively small proportion of 24-h angiography in previous meta-analysis (8), we conducted a systemic review and meta-analysis of randomized controlled trials (RCTs) and observational studies to compare PIT and pPCI in terms of reperfusion efficacy and safety.

## Materials and Methods

This study adheres to the reporting guidelines set by Preferred Reporting Items for Systematic Reviews and Meta-Analysis (PRISMA). In our study, PIT was defined as intravenous thrombolysis in a prehospital setting or at a non-PCI-capable hospital and followed rescue PCI in cases of failed fibrinolysis or routine angiography within 2–24 h in cases of successful fibrinolysis. “Ischemia-guided reperfusion” was excluded because patients with successful thrombolysis may not undergo routine angiography and those with failed thrombolysis may be treated with either immediate PCI or repeat thrombolysis. “Facilitated PCI” was also excluded because all patients underwent immediate PCI after thrombolysis, regardless of time-window between fibrinolysis and percutaneous coronary intervention.

A systematic search for RCTs and observational studies published before February 2022 was conducted on Pubmed, Embase, Cochrane library, and Scopus, with detailed retrieval keywords “pharmacoinvasive/pharmaco-invasive/PIT/pharmacoinvasive strategy/fibrinolysis/thrombolysis/fibrinolysis followed by angioplasty/thrombolysis followed by angioplasty/prehospital thrombolytic therapy/prehospital fibrinolytic therapy/fibrinolysis followed by early invasive therapy/thrombolysis followed by early invasive therapy/fibrinolysis followed by percutaneous coronary intervention/thrombolysis followed by percutaneous coronary intervention” and “primary percutaneous coronary intervention/immediate percutaneous coronary intervention/immediate angioplasty/primary angioplasty” and “STEMI/myocardial infarction” in title or abstract terms. We also searched the reference lists of the STEMI management guidelines and previously published systematic reviews and meta-analyses to identify other relevant studies (1–3, 8). Besides, references from the annual scientific session of the American Heart Association, American College of Cardiology and European Society of Cardiology meetings were also searched, but those without formally published articles were ruled out. Study design, baseline characteristics, interventions, and outcomes of included studies were all extracted and presented in Supplementary Tables 1–4.

The primary efficacy outcome was short-term mortality within in-hospital and 30-day period, and other outcomes including 6-month and 12-month mortality, in-hospital and 30-day heart failure, 30-day cardiogenic shock, and reinfarction within in-hospital period, 30 days, and 12 months were also assessed. The primary safety outcome was 30-day trial-defined major bleeding events, and other safety outcomes included in-hospital major bleeding events, in-hospital stroke, and in-hospital intracranial hemorrhage. RCTs and observational studies were respectively analyzed, and combined pooled analysis was also presented. Definitions of endpoints in RCTs and observational studies were presented in Supplementary Tables 5, 6, respectively.

Cochrane Collaboration’s risk of bias tool was used to assess the quality of included RCTs; Robins-I tool was used to determine the quality of included observational studies (9). Statistics analysis was performed using Cochrane Collaboration Review Manager (Rev Man, Version 5.4.0). Risk ratio (RR) and 95% CI were used as summary statistics from RCTs, and odds ratio (OR) and 95% CI were used as summary statistics from observational studies and combined analysis. Publication bias was evaluated by visual inspection of the funnel plot. *I*^2^ was calculated to evaluate heterogeneity between the included studies, and a value <50% was considered acceptable. Random-effect Mantel–Haenszel model was used if heterogeneity was observed, and fixed-effect Mantel–Haenszel model was chosen if not. Forest plots were used to visually assess the results of pooling. PPCI was designated as the exposure while PIT was the control. A *p-*value of <0.05 (two-sided) was considered statistically significant.

## Results

**FIGURE 1:**
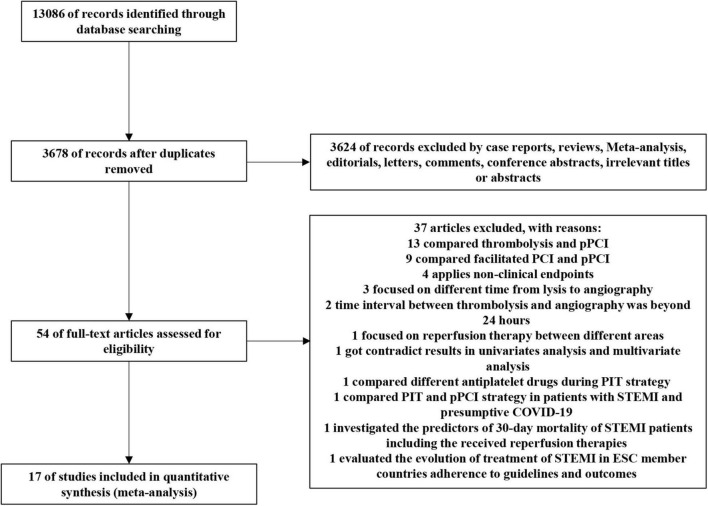
PRISMA flow chart of the selection strategy. PCI, percutaneous coronary intervention; pPCI, primary percutaneous coronary intervention; STEMI, ST-elevation myocardial infarction; COVID-19, Corona Virus Disease 2019; ESC, European Society of Cardiology.

A total of 13,086 potentially relevant reports were retrieved from the initial search of databases. After thorough screening by KL and BiZ, 5 RCTs and 12 observational studies were included for the analysis (Figure 1), representing 26,597 patients receiving pPCI (*n* = 19,912) or PIT (*n* = 6,685).

Among the RCTs, the WEST (Which Early ST-elevation myocardial infarction Therapy) study compared three strategies, such as thrombolysis with tenecteplase, tenecteplase with invasive treatment within 24 h, and pPCI (10); the GRACIA-2 (Grupo de Ana’lisis de la Cardiopatı ‘a Isque’mica Aguda) study compared full-dose tenecteplase followed by stenting within 3–12 h and pPCI within 3 h of randomization (11); the EARLY–MYO trial compared PIT strategy with half-dose alteplase and PCI within 3–24 h versus pPCI (12); two articles came from Strategic Reperfusion Early After Myocardial Infarction (STREAM) study with different follow-up time, comparing PIT strategy with tenecteplase followed by coronary angiography within 6–24 h and pPCI (6, 13). Among the observational studies, the STEPP–AMI study compared PIT strategy with tenecteplase and PCI within 3–24 h versus pPCI (14); the FAST–MI study compared pPCI with PIT, where 78% of patients used tenecteplase and 84% of patients underwent PCI during hospital stay (15); an analysis from the Vital Heart Response (VHR) Program compared pPCI strategy and PIT strategy with tenecteplase, but the median time from successful fibrinolysis to scheduled invasive treatment was 23.4 h (16); the PHASE-MX (Evaluation of Pharmacoinvasive Strategy versus percutaneous coronary intervention in patients with acute myocardial infarction with ST-segment Elevation at the National Institute of Cardiology in Mexico City) study, REPERFUSE Kuwait (reperfusion in ST-elevation myocardial infarction in Kuwait) and another two studies from India and Canada compared PIT with invasive treatment operated within the first 24 h after hospital admission and pPCI in a real-world setting (17–20); Chava et al. compared PIT and pPCI in bleeding complications (21); Bodi et al. compared PIT and pPCI in efficacy through cardiac magnetic resonance change in the first week and sixth month (22); Vincent Auffret compared PIT and pPCI in older patients (age >70 years old) (23); Doo Sun Sim et al. operated a propensity score–matched analysis to evaluate 12-month clinical outcome of STEMI patients undergoing PIT and pPCI from KAMIR (Korea Acute Myocardial Infarction Registry) (24); another study published in last year compared outcomes of timely pPCI (≤120 min), delayed pPCI (121–180 min), late pPCI (>180 min) with PIT strategies (25).

**FIGURE 2:**
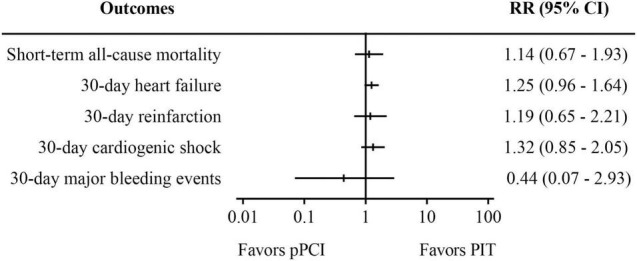
Meta-analysis results of randomized controlled trials. PIT, pharmaco-invasive therapy; pPCI, primary percutaneous coronary intervention.

**FIGURE 3:**
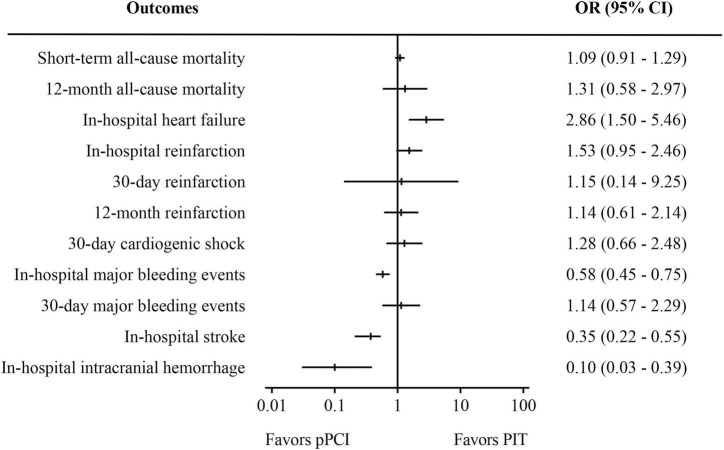
Meta-analysis results of observational studies. PIT, pharmaco-invasive therapy; pPCI, primary percutaneous coronary intervention.

**FIGURE 4:**
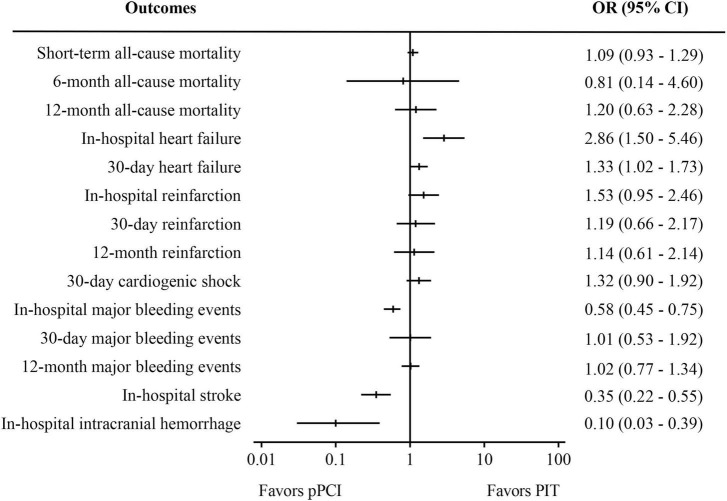
Meta-analysis results of pooled analysis of randomized controlled trials and observational studies. PIT, pharmaco-invasive therapy; pPCI, primary percutaneous coronary intervention.

Supplementary Tables 1–4 summarized the baseline characteristics and important timepoints of the included RCTs and observational studies. All RCTs and observational studies were of considerably high methodologic quality, as seen in Supplementary Figures 1, 2. Blinding was not possible in RCTs due to the nature of the studies, and this was a potential source of bias across all trials. In this meta-analysis, there was no evidence of small study effects or publication bias, with represented funnel plot exhibited as Supplementary Figure 3. Figures 2–4 displays the summarized results of our meta-analysis. Detailed forest plots showing the effect size and weight of each study can be found in Supplementary Figures 4–6. The follow-up time ranged from in-hospital to 12 months. Median time from symptom onset to pPCI ranged from 176 to 342 min, and the median time from symptom onset to fibrinolysis time ranged from 99 to 245 min. However, some important timepoints were not presented in original papers as summarized in Supplementary Tables 2, 4.

### Analysis of Randomized Controlled Trials

Pooled analysis of five RCTs (Figure 2) suggests that there was no significant difference in both short-term all-cause mortality (RR: 1.14, 95% CI: 0.67–1.93, *I*^2^ = 0%, *p* = 0.64) and 30-day major bleeding events (RR: 0.44, 95% CI: 0.07–2.93, *I*^2^ = 0%, *p* = 0.39) between pPCI group and PIT group. Besides, no difference was found regarding 30-day heart failure, reinfarction, and cardiogenic shock between the groups.

### Analysis of Observational Studies

Pooled analysis of 12 observational studies (Figure 3) showed that there was no significant difference in both short-term all-cause mortality (OR: 1.09, 95% CI: 0.91–1.29, *I*^2^ = 0%, *p* = 0.35) and 30-day major bleeding events (OR: 1.14, 95% CI: 0.57–2.29, *I*^2^ = 0%, *p* = 0.71). However, pPCI significantly reduced risk of in-hospital stroke (OR: 0.35, 95% CI: 0.22–0.55, *I*^2^ = 0%, *p* < 0.01), in-hospital major bleeding events (OR: 0.58, 95% CI: 0.45–0.75, *I*^2^ = 42%, *p* < 0.01), and in-hospital intracranial bleeding (OR: 0.10, 95% CI: 0.03–0.39, *I*^2^ = 17%, *p* < 0.01), but significantly increased risk of in-hospital heart failure (OR: 2.86, 95% CI: 1.50–5.46, *I*^2^ = 0%, *p* < 0.01). There was no significant difference in 12-month mortality, 30-day cardiogenic shock, and reinfarction within in-hospital period, 30 days and 12 months.

### Combined Analysis of Randomized Controlled Trials and Observational Studies

Combined results of RCTs and observational studies (Figure 4) were partly similar to the results of analysis of the observational studies, considering the relatively small proportion of RCTs. There was no significant difference in short-term all-cause mortality (OR: 1.09, 95%CI 0.93-1.29, I^2^ = 0%, *p* = 0.30) Compared with PIT, pPCI significantly reduced risk of in-hospital stroke (OR: 0.35, 95% CI: 0.22–0.55, *I*^2^ = 0%, *p* < 0.01) and in-hospital intracranial hemorrhage (OR: 0.10, 95% CI: 0.03–0.39, *I*^2^ = 17%, *p* < 0.01), while it significantly increased risk of in-hospital heart failure (OR: 2.86, 95% CI: 1.50–5.46, *I*^2^ = 0%, *p* < 0.01) and 30-day heart failure (OR: 1.33, 95% CI: 1.02–1.73, *I*^2^ = 0%, *p* = 0.04). There was no difference in 6- and 12-month mortality, 30-day cardiogenic shock and reinfarction within in-hospital period, 30 days, and 12 months between the two groups. Regarding safety outcomes, the rate of in-hospital major bleeding events was significantly lower in the pPCI group (OR: 0.58, 95% CI: 0.45–0.75, *I*^2^ = 42%, *p* < 0.01), though the difference was not observed in rates of 30-day (OR: 1.01, 95% CI: 0.53–1.92, *I*^2^ = 0%, *p* = 0.98) and 12-month major bleeding events.

## Discussion

Our meta-analysis of RCTs and combined analysis of RCTs and observational studies suggests no significant difference in all-cause mortality and major bleeding events within 30 days after reperfusion with PIT or pPCI strategy in STEMI patients. Though an observational study published in 2016 showed that the PIT was associated with reduced mortality compared to pPCI in univariate analysis, the difference disappeared after adjusting baseline covariates (26). Interestingly, our combined analysis showed that pPCI was associated with reduced risk of in-hospital major bleeding events, stroke and intracranial bleeding, but higher risk of in-hospital and 30-day heart failure. Another meta-analysis comparing pPCI and PIT showed similar all-cause short-term mortality risk with reduced risks of total stroke, hemorrhagic stroke, reinfarction, and increased risk of cardiogenic shock in pPCI group (8). However, the proportion of 24-h angiography in that meta-analysis is relatively small, though insignificant difference in mortality, reinfarction and total stroke favored PIT therapy in sensitivity analysis of mandatory 24-h angiography. Studies included in the current analysis were more likely to comply with current guidelines-defined PIT, which could explain the outcome differences between the meta-analyses.

Considering mortality advantage within the time-window (27, 28), current guidelines preferred pPCI as a better option to reperfusion than thrombolysis when PCI-related delay was expected to be longer than 120 min (1–3), while system delay and patient delay make it harder to control the total ischemic time in real world. Previous studies showed that reduction of system delay was associated with reduced mortality among patients with STEMI treated with pPCI (4, 29–31), and another randomized study found that a shorter system delay resulted in smaller infarct size, larger myocardial salvage index, and improved left function in cardiac magnetic resonance in patients treated with pPCI (32). Among components of system delays, inter-hospital transfers occupied the majority (33). A nationwide analysis showed that more than 1/3 of the United States STEMI patients requiring transference for pPCI failed to achieve door-to-device time of shorter than 120 min, despite estimated transfer time <60 min (34). And thus, programs focusing on reducing system delays were operated to improve the quality of care for patients with STEMI. Mission: Lifeline program reduced time from first medical contact to emergency medical systems transport to PCI-capable hospitals, time from first door to device for transfers for pPCI, and time from door-in to door-out at non-PCI-capable hospitals in the United States hospitals (35). In China, barriers associated with delay in STEMI treatment were identified at both patient and system levels (36); specifically speaking, less likely to experience early presentation, longer onset-to-arrival time, lower rate of reperfusion therapy and higher rate of in-hospital mortality were commonly seen in prefecture-level or county-level hospitals (37). Thus, China chest pain center (CPC) accreditation program was officially initiated in 2013 to promote the management of patients with acute chest pain by establishing a regional emergency care network (38), which significantly reduced time delays and improved in-hospital outcomes for acute myocardial infarction patients (39–41). However, the risk of major adverse cardiovascular events and all-cause death both followed a reverse *J*-shaped trend: the risk of major adverse cardiovascular events and all-cause death decreased gradually after achieving CPC accreditation, but increased in the second year (39). Thus, China CPC is also looking for another reperfusion strategy for STEMI patients who cannot accept pPCI treatment in time. PIT, which combined thrombolysis and PCI, could be an important complement for pPCI. According to China CPC’s STEMI protocol, delays in treatment timelines and increased in-hospital mortality and heart failure are witnessed during coronavirus disease-19 outbreak in China, especially in Hubei province; the outbreak had a substantial positive effect on the probability of thrombolysis compared to pPCI, but insignificant difference of effective reperfusion rate was found between the two (42). A recent analysis from Norway also showed that PIT strategy was associated with better long-term survival in STEMI patients who did not receive timely pPCI (25). In our analysis, PIT shared similar efficacy with pPCI regarding short-term mortality, which could be explained by earlier reperfusion treatment with thrombolytic agents in PIT group. In an observational study, the median time from symptom onset to reperfusion treatment reached 170 min earlier in the PIT group than pPCI group (15).

Despite the insignificant difference in mortality, PIT was found to reduce in-hospital and 30-day heart failure risk in our analysis. Previous studies have proved that new-onset heart failure was associated with delay in time to reperfusion (43–45), and thus, the decreased time delay of thrombolysis in PIT group may contribute to less new-onset heart failure compared with pPCI. Besides the epicardial coronary artery reperfusion, some studies focus on microvascular perfusion and cardiac function recovery between pPCI and PIT. Shavadia et al. analyzed peak cardiac biomarker in patients from STREAM trial who were randomized to receive PIT or pPCI, showing that PIT resulted in fewer large infarct and more medium-sized ones (46); similarly, Pu et al. also found that cardiac MRI-defined infarct size, left ventricular ejection fraction, and incidence of microvascular obstruction were similar between PIT group and pPCI group at a median of 5 days post myocardial infarction in EARLY–MYO trial (12). Therefore, in addition to decreased time delay, better microvascular perfusion with PIT may also contribute to the reduced risk of incident heart failure. Previous meta-analysis indicated that PIT reduced the risk of cardiogenic shock (8), but the benefit was not observed in our analysis, primarily due to different inclusion criteria between the two studies. In our study, we included subgroups who underwent routine angiography after successful thrombolysis but not those who underwent only usual care.

Despite the mentioned advantages, bleeding is always the greatest drawback to PIT. Notwithstanding the significant difference of 30-day major bleeding events between the two groups was not found in our analysis, in-hospital major bleeding events and intracranial hemorrhage were still large concerns. Among studies included in our analysis, two observational studies contributed to the major difference in stroke, especially hemorrhagic apoplexy (20, 23). Considering similar thrombolytic agents use and concomitant antithrombotic therapy of the two studies, the authors thought that the increased risk was driven by two main processes: (i) older age of participants in the two studies from the baseline characteristics and (ii) full-dose thrombolytic agents were used in the two studies, which increased the risk of bleeding. According to current guidelines, a half-dose of thrombolytic agent should be considered in patients over 75 years of age, or even in some study, all participants received half-dose thrombolytic agents regardless of age, but the efficacy and safety outcomes between pPCI and PIT still turned out satisfying (12). Thus, there remained some unanswered questions in PIT, especially the thrombolysis part, which is the major resource of safety concern. With more thrombolytic agents invented, in what kind and dose of thrombolytic agents can we improve pre-PCI Thrombolysis in Myocardial Infarction grade but not at higher risk of bleeding should be investigated. Additionally, age ≥75 years was an independent factor that entailed a 3.5-fold higher major adverse cardiac events and 2-fold higher mortality rate compared to patients <75 years of age in patients with STEMI undergoing PIT (47), and so, research aiming at recognizing population are also warranted. Satisfyingly, the ongoing Second Strategic Reperfusion Early After Myocardial Infarction (STREAM-2) study are supposed to answer the questions above and improve the application of PIT strategy in the real world. Though PIT strategy was found non-inferior to pPCI strategy regarding short-term mortality and major bleeding events in our analysis, previous studies found that proper time interval between thrombolysis and PCI was the major determinant (8, 48), and thus, PIT strategy could be a complement for situations where timely pPCI was not possible, such as long-distance transfer (34) and logistic delays by social background (42) or medical institution (37).

There are several limitations in our analysis. First, important information was not clarified in some included studies, and thus, heterogeneity between rescue-PCI proportion, definitions of endpoints, time from symptom onset to treatment in both groups and time from randomization/lysis to routine early PCI in PIT group among the included studies might influence the major results in the present analysis. Second, there was relatively low weight of RCTs, because the operation of such trials is not easy in the era of pPCI; therefore, more large-scale RCTs are urgently warranted. Third, the inclusion of observational studies could be a source of selection bias and unknown confounders because of the lack of randomization. Last, differences in patient selection criteria across the included studies could have contributed to bias.

## Conclusion

No significant difference was observed in short-term mortality and major bleeding events between pPCI strategy and PIT strategy. PIT could be an important complement for pPCI in real world in selected patients under specific conditions. However, more large-scale RCTs are warranted to further validate its efficacy and safety.

## Data Availability Statement

The original contributions presented in the study are included in the article/Supplementary Material, further inquiries can be directed to the corresponding authors.

## Author Contributions

KL and BiZ collected the data and drafted the manuscript. BoZ, YZ, and YH were in charge of the study concept and design and helped with result interpretations. All authors contributed to the article and approved the submitted version.

## Conflict of Interest

The authors declare that the research was conducted in the absence of any commercial or financial relationships that could be construed as a potential conflict of interest.

## Publisher’s Note

All claims expressed in this article are solely those of the authors and do not necessarily represent those of their affiliated organizations, or those of the publisher, the editors and the reviewers. Any product that may be evaluated in this article, or claim that may be made by its manufacturer, is not guaranteed or endorsed by the publisher.

## Supplementary Material

Supplementary Figure 1 Results of risk of bias assessment for included randomized controlled trials using Cochrane Collaboration’s risk of bias tool.

Supplementary Figure 2 Results of risk of bias assessment for included observational studies using Robins-I tool.

Supplementary Figure 3 Represented funnel plot to assess publication bias. This representative funnel plot came from pooled analysis for short-term all-cause mortality using combined data of randomized controlled trials and observational studies.

Supplementary Figure 4 Detailed forest plots of pooled analysis of included randomized controlled trials. PIT: pharmaco-invasive therapy; pPCI: primary percutaneous coronary intervention.

Supplementary Figure 5 Detailed forest plots of pooled analysis of included observational studies. PIT: pharmaco-invasive therapy; pPCI: primary percutaneous coronary intervention.

Supplementary Figure 6 Detailed forest plots of combined analysis of included randomized controlled trials and observational studies. PIT: pharmaco-invasive therapy; pPCI: primary percutaneous coronary intervention.

Supplementary Table 1 Baseline characteristics and important information of included randomized controlled trials. ^†^Data of “pharmaco-invasive strategy” group were described according to “rescue angiography” and “scheduled angiography,” respectively. “A/B” means “data in pPCI/data in PIT”. DM, diabetes mellitus; HTN, hypertension; MI, myocardial infarction; PCI, percutaneous coronary intervention; PIT, pharmaco-invasive therapy; pPCI, primary percutaneous coronary intervention; GPI, Glycoprotein IIb/IIIa receptor inhibitors; NA, not mentioned.

Supplementary Table 2 Important timepoints of included randomized controlled trials. ^†^Interval from onset of symptoms to randomization + Interval from randomization to angiography. ^‡^Rescue angiography/Rescue angiography. PCI, percutaneous coronary intervention; PIT, pharmaco-invasive therapy; pPCI, primary percutaneous coronary intervention; NA, not mentioned.

Supplementary Table 3 Baseline characteristics and important information of included observational studies. “A/B” means “data in pPCI/data in PIT.” *Data of pPCI were expressed in groups of timely pPCI (≤120 min), delayed pPCI (121–181 min) and late pPCI (>180 min). DM, diabetes mellitus; HTN, hypertension; MI, myocardial infarction; PCI, percutaneous coronary intervention; PIT, pharmaco-invasive therapy; pPCI, primary percutaneous coronary intervention; GPI, Glycoprotein IIb/IIIa receptor inhibitors; NA, not mentioned.

Supplementary Table 4 Important timepoints of included observational studies. ^†^Time from onset of symptoms to arrival at first hospital + Time from arrival at first hospital to administration of fibrinolytics. ^‡^Symptom-onset to door time + Door to balloon time. *“A/B/C” indicates: A: data from timely pPCI (≤120 min); B: data from delayed pPCI (121–181 min); C: data from late pPCI (>180 min). PCI, percutaneous coronary intervention; PIT, pharmaco-invasive therapy; pPCI, primary percutaneous coronary intervention; NA, not mentioned.

Supplementary Table 5 Outcome definitions of included randomized controlled trials.

Supplementary Table 6 Outcome definitions of included observational studies.

## Abbreviations

PIT: pharmaco-invasive therapy
PCI: percutaneous coronary intervention
pPCI: primary percutaneous coronary intervention
RCTs: randomized controlled trials
RR: risk ratio
OR: odds ratio
STEMI: ST-elevation myocardial infarction
CPC: chest pain center.
