# Supplementary figures and images for: Reperfusion Strategy of ST-Elevation Myocardial Infarction: A Meta-Analysis of Primary Percutaneous Coronary Intervention and Pharmaco-Invasive Therapy
Source: Front Cardiovasc Med. 2022 Mar 17;9:813325. doi: 10.3389/fcvm.2022.813325 (PMC8970601; doi:10.3389/fcvm.2022.813325)

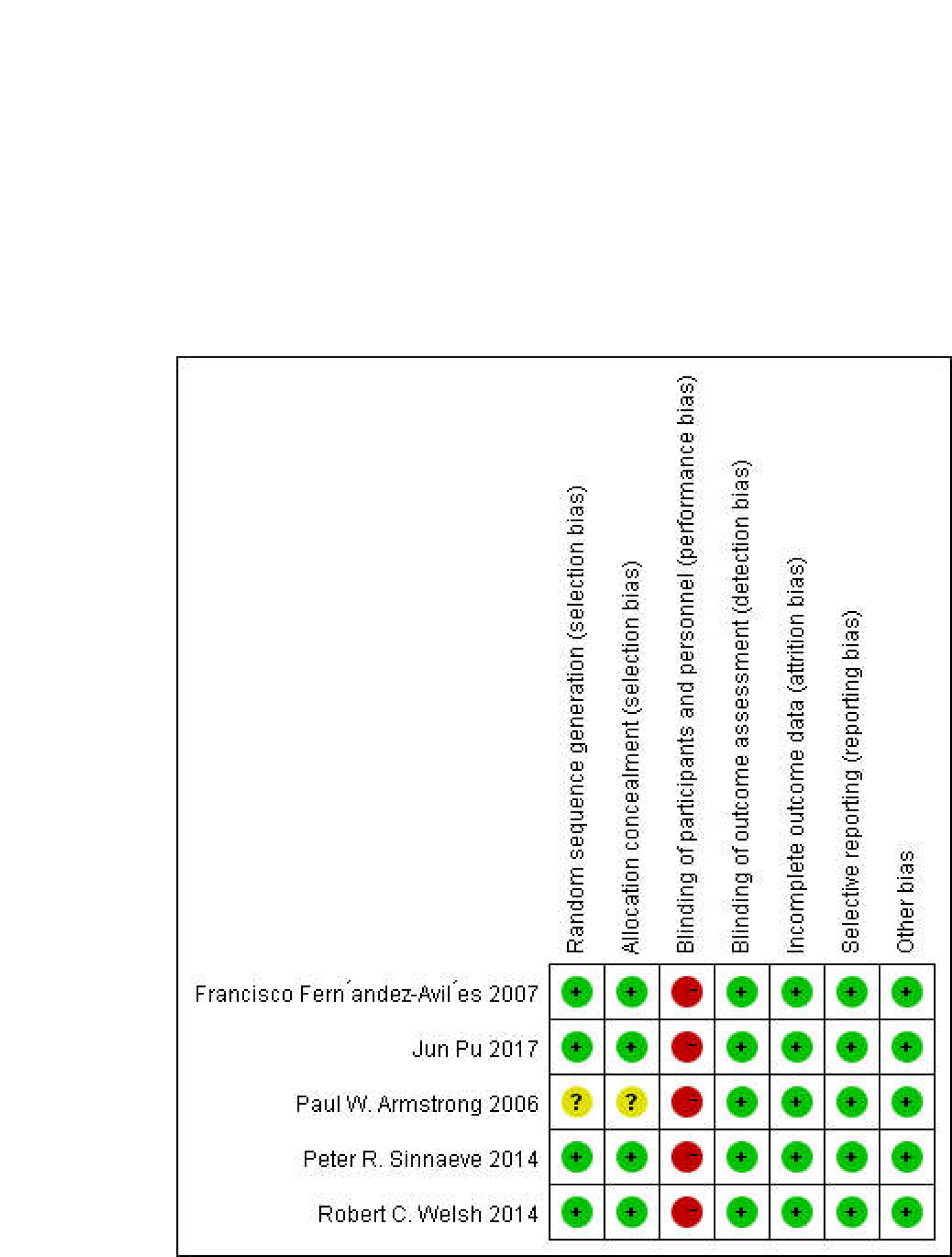

Supplement: Supplementary Figure 1 — Results of risk of bias assessment for included randomized controlled trials using Cochrane Collaboration’s risk of bias tool. [file Image_1.TIF]

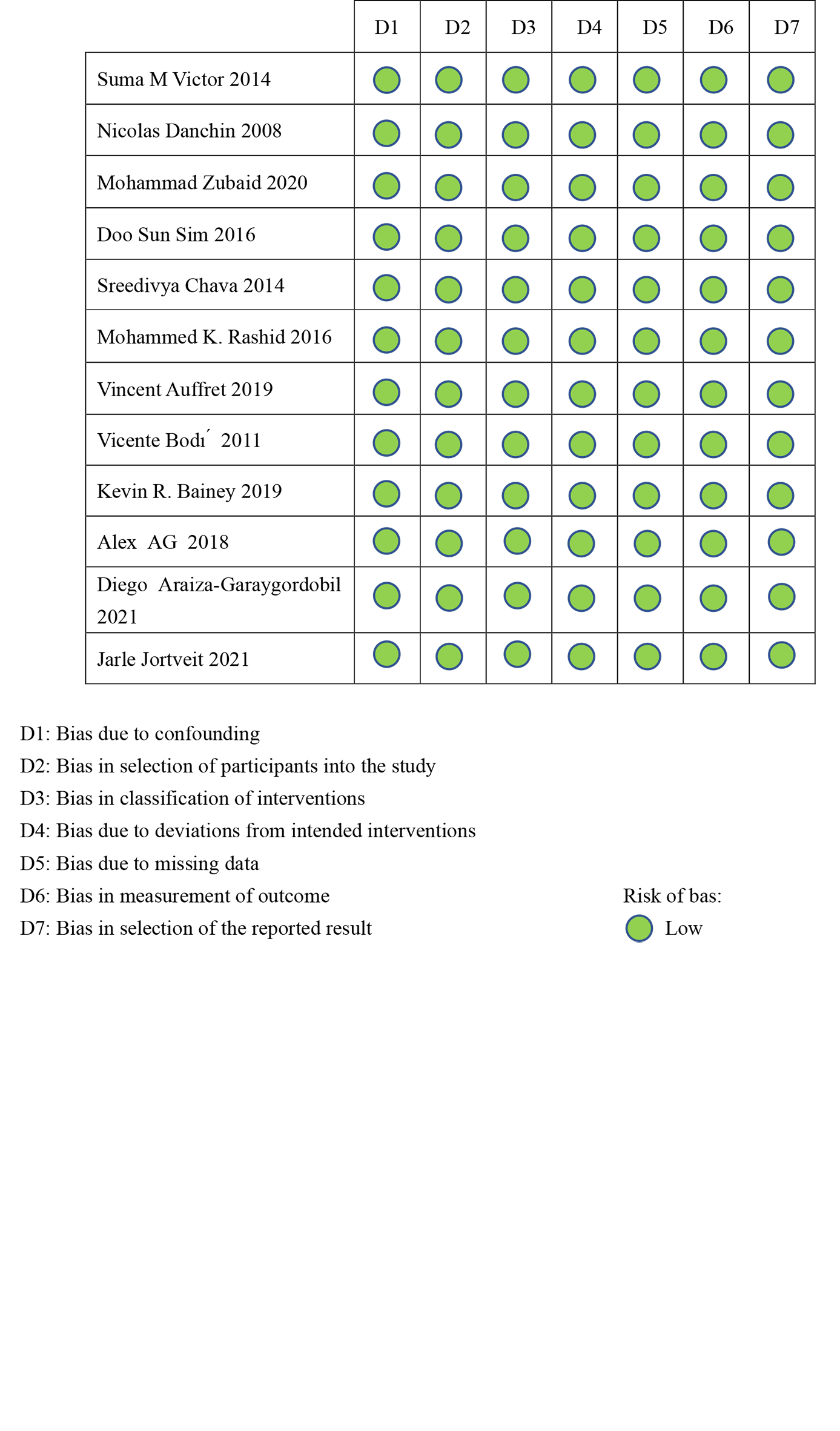

Supplement: Supplementary Figure 2 — Results of risk of bias assessment for included observational studies using Robins-I tool. [file Image_2.TIF]

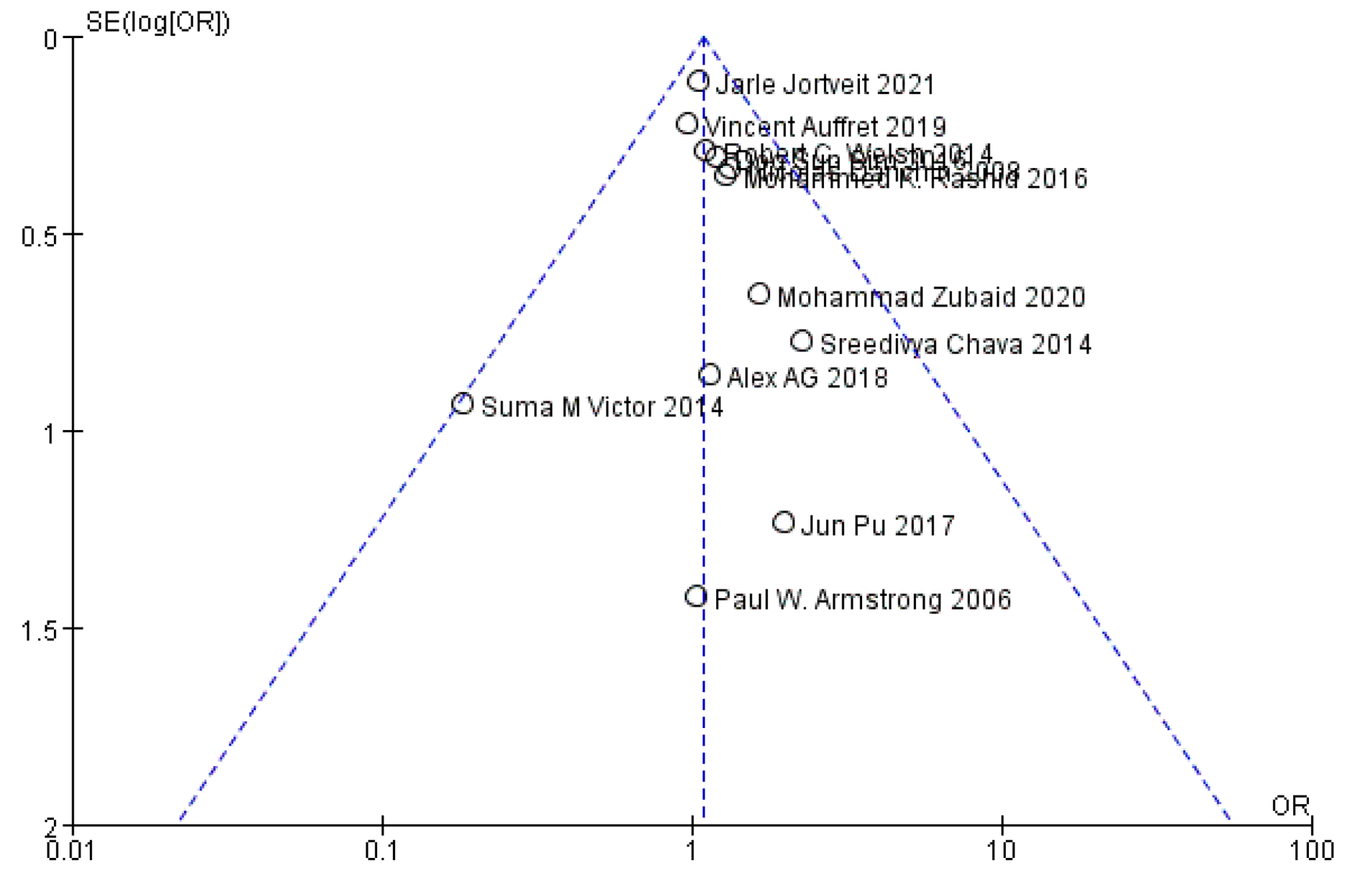

Supplement: Supplementary Figure 3 — Represented funnel plot to assess publication bias. This representative funnel plot came from pooled analysis for short-term all-cause mortality using combined data of randomized controlled trials and observational studies. [file Image_3.TIF]

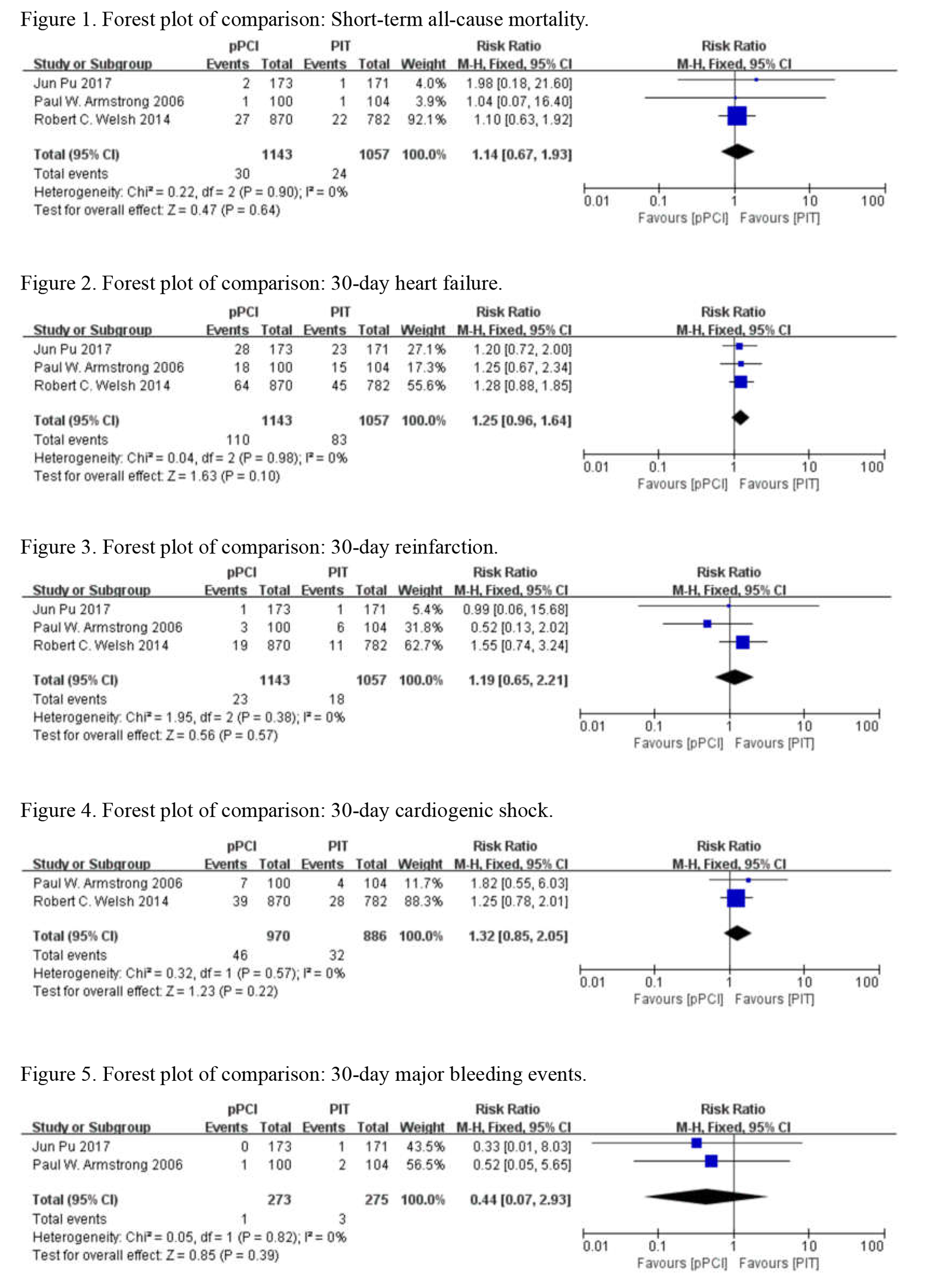

Supplement: Supplementary Figure 4 — Detailed forest plots of pooled analysis of included randomized controlled trials. PIT: pharmaco-invasive therapy; pPCI: primary percutaneous coronary intervention. [file Image_4.TIF]

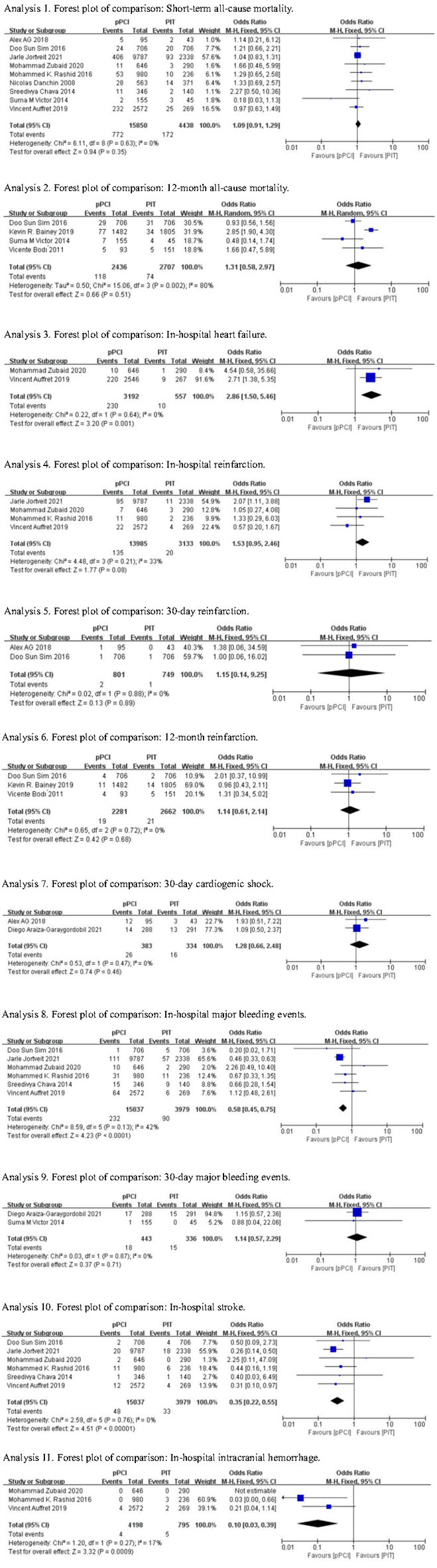

Supplement: Supplementary Figure 5 — Detailed forest plots of pooled analysis of included observational studies. PIT: pharmaco-invasive therapy; pPCI: primary percutaneous coronary intervention. [file Image_5.TIFF]

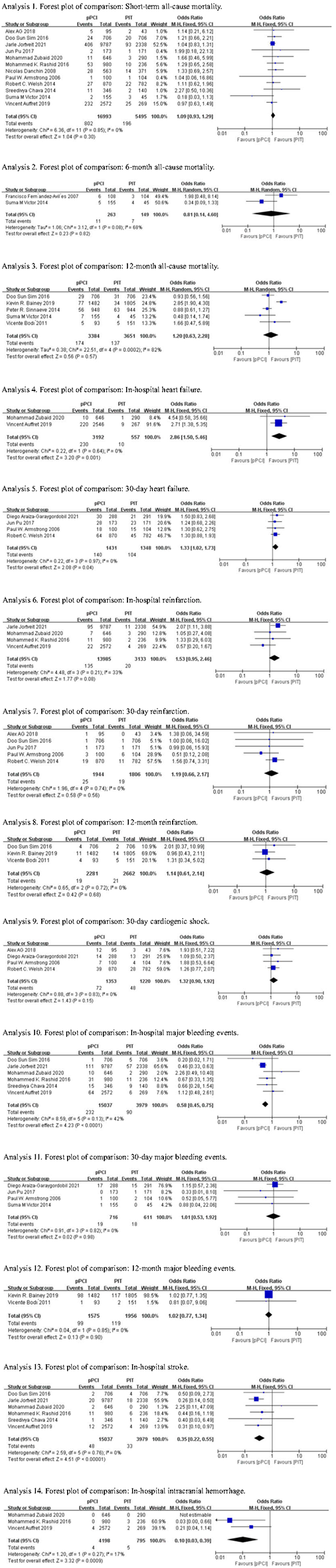

Supplement: Supplementary Figure 6 — Detailed forest plots of combined analysis of included randomized controlled trials and observational studies. PIT: pharmaco-invasive therapy; pPCI: primary percutaneous coronary intervention. [file Image_6.TIFF]
