# Supplementary material for: Reperfusion Strategy of ST-Elevation Myocardial Infarction: A Meta-Analysis of Primary Percutaneous Coronary Intervention and Pharmaco-Invasive Therapy
Source: Front Cardiovasc Med. 2022 Mar 17;9:813325. doi: 10.3389/fcvm.2022.813325 (PMC8970601; doi:10.3389/fcvm.2022.813325)
Supplement: Supplementary Table 1 — Baseline characteristics and important information of included randomized controlled trials. †Data of “pharmaco-invasive strategy” group were described according to “rescue angiography” and “scheduled angiography,” respectively. “A/B” means “data in pPCI/data in PIT”. DM, diabetes mellitus; HTN, hypertension; MI, myocardial infarction; PCI, percutaneous coronary intervention; PIT, pharmaco-invasive therapy; pPCI, primary percutaneous coronary intervention; GPI, Glycoprotein IIb/IIIa receptor inhibitors; NA, not mentioned. [file Table_1.DOCX]

**Table S1. Baseline characteristics and important information of included randomized controlled trials.**

| Study | Total Participants | Follow-up time | Male % | Mean Age | DM % | HTN % | Anterior MI % | Rescue PCI/PIT | Fibrinolytic agents | P2Y12 inhibitors | GPI |
| --- | --- | --- | --- | --- | --- | --- | --- | --- | --- | --- | --- |
| Armstrong, 2006 | 100/104 | 30 days | 78%/81.7% | 60/57 | 16.0%/7.7% | 33.0%/53.8% | 42.0%/33.6% | 29/104 | Tenecteplase | Clopidogrel | Recommended for all PCI procedures unless within 3h of fibrinolytic therapy |
| Fern ́andez-Avil ́es, 2006 | 108/104 | 6 months | 82.4%/79.8% | 64.3±12.9/ 62.5±12.6 | 27.8%/23.1% | 39.8%/41.3% | 45.6%/47.7% | 3/104 | Tenecteplase | NA | Frequently given to patients who were assigned pPCI group; operator discretion in PIT group |
| Welsh, 2014 | 870/782 | 30 days | 78.1%/NA† | 59/NA† | 13.0%/NA† | 44.1%/NA† | 45.3%/NA† | 318/782 | Tenecteplase | Clopidogrel | NA |
| Sinnaeve, 2014 | 948/944 | 12 months | 78.1%/79.4% | 59.6±12.5/59.7±12.4 | 13.1%/12.1% | NA | 45.6%/48.1% | NA | Tenecteplase | Clopidogrel | Additional discretionary |
| Pu, 2017 | 173/171 | 30 days | 88.6%/89.4% | 58 (50‒64)/59 (52‒65) | 25.6%/24.8% | 49.7%/53.4% | 52.7%/49.1% | 41/171 | Alteplase (half-dose) | Clopidogrel/Ticagrelor | Not allowed in any patient before PCI but was permitted during or after catheterization at the investigator’s discretion |

† Data of 'pharmaco-invasive strategy' group were described according to 'rescue angiography' and 'scheduled angiography' respectively. “A/B” means “data in pPCI/data in PIT”. Abbreviations: DM: diabetes mellitus; HTN: hypertension; MI: myocardial infarction; PCI: percutaneous coronary intervention; PIT: pharmaco-invasive therapy; pPCI: primary percutaneous coronary intervention; GPI: Glycoprotein IIb/IIIa receptor inhibitors; NA: not mentioned.
