# Supplementary material for: Reperfusion Strategy of ST-Elevation Myocardial Infarction: A Meta-Analysis of Primary Percutaneous Coronary Intervention and Pharmaco-Invasive Therapy
Source: Front Cardiovasc Med. 2022 Mar 17;9:813325. doi: 10.3389/fcvm.2022.813325 (PMC8970601; doi:10.3389/fcvm.2022.813325)
Supplement: Supplementary Table 2 — Important timepoints of included randomized controlled trials. †Interval from onset of symptoms to randomization + Interval from randomization to angiography. ‡Rescue angiography/Rescue angiography. PCI, percutaneous coronary intervention; PIT, pharmaco-invasive therapy; pPCI, primary percutaneous coronary intervention; NA, not mentioned. [file Table_2.DOCX]

**Table S2. Important timepoints of included randomized controlled trials.**

| Study | Onset to treatment (min) | | Onset to PCI (PIT) | Onset to rescue PCI (min) (PIT) | Failed thrombolysis to PCI (PIT) | Onset to non-rescue PCI (min) (PIT) | Time from randomization/lysis to routine early PCI (min) |
| --- | --- | --- | --- | --- | --- | --- | --- |
|  | pPCI | PIT |  |  |  |  |  |
| Armstrong, 2006 | 176 (140-280) | 130 (75-185) | 425 (288– 1331) | 277 (213–381) | NA | 926 (398-1454) | NA |
| Fern ́andez-Avil ́es, 2006 | 180 (120-240) + 60 (42-84) † | NA | 180 (120-240) + 276 (204-486) † | NA | NA | NA | 276 (204-486) |
| Welsh, 2014 | 177 (135-228) | 99 (75-145) / 100 (75-140) ‡ | NA | 88 (66-135) + 140 (114-172) † | NA | 91 (68-130) + 1101 (768-1333) † | 1101 (768-1333) |
| Sinnaeve, 2014 | NA | NA | NA | NA | NA | NA | NA |
| Pu, 2017 | 280 (214–340) | 210 (166–270) | 695 (451–1115) | NA | NA | NA | 521 (303–957) |

† Interval from onset of symptoms to randomization + Interval from randomization to angiography. ‡ Rescue Angiography / Rescue Angiography. Abbreviations: PCI: percutaneous coronary intervention; PIT: pharmaco-invasive therapy; pPCI: primary percutaneous coronary intervention; NA: not mentioned.
