# Supplementary material for: Reperfusion Strategy of ST-Elevation Myocardial Infarction: A Meta-Analysis of Primary Percutaneous Coronary Intervention and Pharmaco-Invasive Therapy
Source: Front Cardiovasc Med. 2022 Mar 17;9:813325. doi: 10.3389/fcvm.2022.813325 (PMC8970601; doi:10.3389/fcvm.2022.813325)
Supplement: Supplementary Table 3 — Baseline characteristics and important information of included observational studies. “A/B” means “data in pPCI/data in PIT.” *Data of pPCI were expressed in groups of timely pPCI (≤120 min), delayed pPCI (121–181 min) and late pPCI (>180 min). DM, diabetes mellitus; HTN, hypertension; MI, myocardial infarction; PCI, percutaneous coronary intervention; PIT, pharmaco-invasive therapy; pPCI, primary percutaneous coronary intervention; GPI, Glycoprotein IIb/IIIa receptor inhibitors; NA, not mentioned. [file Table_3.DOCX]

**Table S3. Baseline characteristics and important information of included observational studies.**

| Study | Total Participants | Follow-up time | Male % | Mean Age | DM % | HTN % | Anterior MI % | Rescue PCI in PIT | Fibrinolytic agents | P2Y12 inhibitors in PIT | GPI |
| --- | --- | --- | --- | --- | --- | --- | --- | --- | --- | --- | --- |
| Danchin, 2008 | 563/371 | 30 days | 74%/78% | 61.9 ± 14.1/60.6 ± 12.8 | 19%/14% | 47%/41% | 40%/35% | 144/371 | Tenecteplase: 78%; other agents for remaining cases | Clopidogrel | 68% in pPCI group; 16% in PIT group |
| Bodı ́, 2011 | 93/151 | 12 months | 81%/85% | 60 ±13/58 ± 11 | 15%/15% | 46%/44% | 51%/57% | 35/151 | Tenecteplase | Clopidogrel | NA |
| Chava, 2014 | 346/140 | During hospitalization | 74.9%/65.7% | 62.4 ± 12.9/61.5 ± 12.5 | 20.2%/27.1% | 60.7%/59.3% | 30.6%/34.3% | 78/140 | NA | Clopidogrel | At the discretion of the operator |
| Victor, 2014 | 155/45 | 30 days | 86.5%/86.7% | 54/54 | 50.3%/53.3% | 30.3%/31.1% | NA | 4/45 | Tenecteplase | Clopidogrel | 43.2% in pPCI group; 6.7% in PIT group |
| Rashid, 2016 | 980/236 | During hospitalization | 72.4%/74.6% | 62.7 ± 13.3/61.2 ± 11.6 | 16.4%/20.9% | 47.7%/49.6% | 40.6%/33.9% | NA | Tenecteplase | Clopidogrel | NA |
| Sim, 2016 | 706/706 | During hospitalization/30 days/12 months | 82.0%/80.7% | 58.2±13.1/58.5±11.5 | 21.5%/20.7% | 39.4%/42.2% | 50.8%/49.7% | 271/706 | Tenecteplase:51.6%/Alteplase:40.8%/Urokinase:7.6% | Clopidogrel | 18.6% in pPCI group; 7.9% in PIT group |
| AG, 2018 | 95/43 | 30 days | 93.7%/83.7% | 57.6±12.2/52.7±14.3 | 40.0%/30.2% | 39.4%/42.2% | 50.8%/49.7% | NA | Streptokinase | NA | NA |
| Auffret, 2019 | 2572/269 | During hospitalization | 63.6%/66.2% | 78/76 | 11.3%/12.4% | 59.7%/53.2% | 43.1%/44.2% | 86/213 | Tenecteplase:96%; Reteplase:4% | Clopidogrel:97.0%; Ticagrelor:1.9%; Prasugrel:1.9% | NA |
| Bainey, 2019 | 1482/1805 | 12 months | 75.6%/70.9% | 60/58 | 18.5%/16.2% | 47.8%/43.9% | 41.1%/52.5% | 493/1805 | Tenecteplase | Clopidogrel/Ticagrelor: 98.6% | 63.2% in pPCI group; 25.4% in PIT group |
| Zubaid, 2020 | 646/290 | During hospitalization | 93.7%/94.1% | 53.7/52.3 | 31.7%/34.8% | 38.5%/38.6% | 52.9%/50.7% | 22/290 | Tenecteplase/Reteplase | NA | NA |
| Araiza-Garaygordobil, 2021 | 288/291 | 30 days | 87.2%/86.9% | 59.7±10.8/57.3±10.9 | 31.2%/40.6% | 47.2%/42.9% | 42.0%/42.2% | 120/291 | Tenecteplase: 77.0%; Alteplase: 21.9%; Streptokinase: 1.0% | Clopidogrel:92%; Ticagrelor/Prasugrel:0.03% | 3.1% in pPCI group; not mentioned in PIT group |
| Jarle Jortveit, 2021 | (7238,1537,1012)/2338* | During hospitalization | (79.6%,72.3%,71.8%)/78.0%* | (63.3±11.9,65.7±12.9,65.9±12.8)/62.5±11.1* | (12.2%,16.4%,17.8%)/11.3%* | NA | NA | NA | NA | NA | NA |

“A/B” means “data in pPCI/data in PIT”. *Data of pPCI were expressed in groups of timely pPCI (≤120min), delayed pPCI (121-181min) and late pPCI (＞180min). Abbreviations: DM: diabetes mellitus; HTN: hypertension; MI: myocardial infarction; PCI: percutaneous coronary intervention; PIT: pharmaco-invasive therapy; pPCI: primary percutaneous coronary intervention; GPI: Glycoprotein IIb/IIIa receptor inhibitors; NA: not mentioned.
