# Supplementary material for: Reperfusion Strategy of ST-Elevation Myocardial Infarction: A Meta-Analysis of Primary Percutaneous Coronary Intervention and Pharmaco-Invasive Therapy
Source: Front Cardiovasc Med. 2022 Mar 17;9:813325. doi: 10.3389/fcvm.2022.813325 (PMC8970601; doi:10.3389/fcvm.2022.813325)
Supplement: Supplementary Table 4 — Important timepoints of included observational studies. †Time from onset of symptoms to arrival at first hospital + Time from arrival at first hospital to administration of fibrinolytics. ‡Symptom-onset to door time + Door to balloon time. *“A/B/C” indicates: A: data from timely pPCI (≤120 min); B: data from delayed pPCI (121–181 min); C: data from late pPCI (>180 min). PCI, percutaneous coronary intervention; PIT, pharmaco-invasive therapy; pPCI, primary percutaneous coronary intervention; NA, not mentioned. [file Table_4.DOCX]

**Table S4. Important timepoints of included observational studies.**

| Study | Onset to treatment (min) | | Onset to PCI (PIT) | Onset to rescue PCI (min) (PIT) | Failed thrombolysis to PCI (PIT) (min) | Time from randomization/lysis to routine early PCI (min) |
| --- | --- | --- | --- | --- | --- | --- |
|  | pPCI | PIT |  |  |  |  |
| Danchin, 2008 | 300 (200-555) | 130 (90-215) | NA | NA | NA | NA |
| Bodı ́, 2011 | 212 (140-394) | 180 (120-270) | NA | NA | NA | NA |
| Chava, 2014 | NA | NA | NA | NA | NA | NA |
| Victor, 2014 | 260 (185-390) | 245 (185-395) | NA | NA | NA | 735 (270–1420) |
| Rashid, 2016 | 204 (141-312) | 92 (55-147) + 31 (18-60) † | 92 (55-147) + 31 (18-60) † | NA | NA | 260 (210-385) |
| Sim, 2016 | 241 (160–378) | 165 (92–283) | NA | NA | NA | 2406 (522-4554) |
| AG, 2018 | 240 (180-420) + 80 (75-90) ‡ | NA | NA | NA | NA | 600 (300-980) |
| Auffret, 2019 | 227 (165-335) | 180 (115-280) | NA | NA | 138 (110-185) | 1185 (152-2785) |
| Bainey, 2019 | 212 (138-349) | 128 (80-210) | NA | NA | NA | NA |
| Zubaid, 2020 | 198 (132-306) | 113 (60-196) + 35 (23-55) † | 1140 (750-1620) | NA | NA | 996 (570-1350) |
| Araiza-Garaygordobil, 2021 | 120 (60-270) +132 (80-245) ‡ | 117 (60-227) + 120 (59-237) † | NA | NA | 648 (258-1440) | 1320 (360-2880) |
| Jarle Jortveit, 2021 | [54 (26-120)+82 (65-100)]/[74 (30-155)+140 (130-156)]/[90 (33-182)+252 (206-365)] ‡* | 96 (60-160)+48 (32-70) † | NA | NA | NA | 255 (145-833) |

† Time from onset of symptoms to arrival at first hospital + Time from arrival at first hospital to administration of fibrinolytics. ‡ Symptom-onset to door time + Door to balloon time. *“A/B/C” indicates: A: data from timely pPCI (≤120min); B: data from delayed pPCI (121-181min); C: data from late pPCI (＞180min). Abbreviations: PCI: percutaneous coronary intervention; PIT: pharmaco-invasive therapy; pPCI: primary percutaneous coronary intervention; NA: not mentioned.
