# Supplementary material for: Reperfusion Strategy of ST-Elevation Myocardial Infarction: A Meta-Analysis of Primary Percutaneous Coronary Intervention and Pharmaco-Invasive Therapy
Source: Front Cardiovasc Med. 2022 Mar 17;9:813325. doi: 10.3389/fcvm.2022.813325 (PMC8970601; doi:10.3389/fcvm.2022.813325)
Supplement: Supplementary Table 5 — Outcome definitions of included randomized controlled trials. [file Table_5.DOCX]

Table S5. Outcome definitions of included randomized controlled trials.

| Study | Outcome definitions |
| --- | --- |
| Armstrong, 2006 | **Refractory ischemia**: Symptoms of ischemia with ST-deviation or definite T-wave inversion persisting for at least 10 min despite medical management while in hospital.  **Recurrent Myocardial Infarction** (myocardial re-infarction):  I. In the first 18 h after randomization:  a. Recurrent signs and symptoms of ischemia at rest accompanied by new or recurrent ST-segment elevations of >=0.1 mV in at least two contiguous leads lasting>=30 min.  II. After 18 h:  a. New Q-waves (by Minnesota Code Criteria) in two or more leads and/or enzyme evidence of re-infarction: re-evaluation of creatine kinase-MB or troponin to above the upper limit of normal and increased by >50% over the previous value.  b. The total creatine kinase must either be re-elevated to two times or more the upper limit of normal and increased by >25% or be re-elevated to >200 U/mL over the previous value.  1. If re-evaluated to less than two times the upper limit of normal, the total creatine kinase must exceed the upper limit of normal by >50% and exceed the previous value by two-fold or be re-elevated to >200 U/mL.  III. Re-infarction after percutaneous transluminal coronary angioplasty (+/-stenting):  a. Creatine kinase greater than three times the upper limit of normal and 50% greater than the previous value and/or new Q-waves (Minnesota Code) in two or more contiguous leads.  IV. Re-infarction after coronary artery bypass grafting surgery:  a. Creatine kinase greater than five times the upper limit of normal and >=50% greater than the previous value and/or new Q-waves (Minnesota Code) in two or more contiguous leads.  **Congestive heart failure**:  I. Physician's decision to treat congestive heart failure with a diuretic, intravenous inotropic agent or intravenous vasodilator and either   1. the presence of pulmonary edema or pulmonary vascular congestion on chest X-ray believed to be of cardiac cause or 2. at least two of the following: 3. rales greater than one-third up the lung fields believed to be due to congestive heart failure. 4. pulmonary capillary wedge pressure >18 mmHg. 5. dyspnea, with documented pO_2_ less than 80 mmHg on room air or O_2_ saturation <90% on room air, without significant lung disease.   **Cardiogenic shock**: The manifestation of vascular collapse and shock (systolic blood pressure<90 mmHg for at least 30 min or systolic blood pressure>90 mmHg after inotropic or intra-aortic balloon support with a cardiac index <2.2 L/min/m^2^ or <2.5 L/min/m^2^ inotropic or intra-aortic balloon support, peripheral signs of hypoperfusion, and chest X- ray with pulmonary edema.  **Major ventricular arrhythmias**: Ventricular arrhythmias >6 h after randomization requiring electrical cardioversion/defibrillation.  **Major bleeding**: Bleeding that causes hemodynamic compromise requiring blood or fluid replacement, inotropic support, ventricular assist devices, surgical intervention, or cardiopulmonary resuscitation to maintain a sufficient cardiac output. |
| Fern ́andez-Avil ́es, 2006 | **Reinfarction**:  typical chest pain lasting more than 30 min with a new increment of creatine kinase-MB isoenzyme with or without new ECG abnormalities.  The movement of this isoenzyme had to meet the following criteria:  (a) if new chest pain arose within 48 h of initial infarction, creatine kinase MB isoenzyme re-elevation was judged positive when it appeared during the descendent phase of this isoenzyme curve of the initial infarction and reached at least 150% of the last measurement;  (b) if it occurred more than 48 h after the initial infarction, this re-elevation was judged positive when it clearly corresponded to a creatine kinase MB isoenzyme curve before that of the initial infarction and reached a peak at least three times the normal value;  (c) if it occurred within 48 h of angioplasty or surgery, this re-elevation was judged positive when it clearly corresponded to a creatine kinase MB isoenzyme curve before that of the initial infarction and reached a peak at least five times the normal value.  **Myocardial ischemia**:  spontaneous (at rest) or stress-induced recurrence of typical angina pectoris (or anginal equivalent) that had to coincide with new ECG abnormalities, or abnormal stress test.  **Ischemia-driven revascularization**:  any revascularization procedure (percutaneous or surgical) involving any diseased coronary artery after identification of severe myocardial ischemia that had to meet at least one of the following criteria:  (a) spontaneous typical angina (at rest) with ECG changes;  (b) grade III or IV effort angina (Canadian classification);  (c) stress test under blockade showing unequivocal ECG changes, perfusion defects, or regional contractility abnormalities along with one feature of poor prognosis (appearance of ischemia before 100 bpm or 5 METS are reached; functional capacity under 5 METS; hypotension on effort; or ventricular tachycardia on effort).  **Functionally adequate revascularization**:  revascularization of the infarct-related artery plus associated revascularization of all non-culprit lesions with diameter stenosis greater than 90% (visual assessment) threatening areas of viable, functionally important myocardium.  **Major bleeding or vascular complication**:  any complication causing death, need for surgery or transfusion, or extended time in hospital. |
| Welsh, 2014 | **Cardiogenic shock**:  Defined as one of the following:  - Systolic blood pressure <90 mm Hg for at least 30 min (or the need for supportive measures to maintain a systolic blood pressure of >90 mm Hg) in the presence of a heart rate of >60 beat/min in association with signs of end-organ hypoperfusion (cold extremities, low urinary output <30 mL/h and/or mental confusion).  - A cardiac index <2.21 L/(min m^2^) in the presence of a pulmonary capillary wedge pressure of >15 mm Hg.  **Reinfarction**:  In the first 18 h after randomization, reinfarction is defined as recurrent signs and symptoms of ischemia at rest, accompanied by new or recurrent ST-segment elevations of ≥ 0.1 mV in at least 2 contiguous leads lasting ≥30 min  After 18 h, reinfarction is defined as follows:  - New Q waves (by Minnesota Code Criteria) in 2 or more leads and/or enzyme/biochemical evidence of reinfarction: re-elevation of creatine kinase-MB or troponin to greater than the upper limit of normal and increased by ≥50% over the previous value  - If creatine kinase - MB or troponin is not available, the total creatine kinase will be evaluated  - The total creatine kinase must either be reevaluated to ≥2 times the upper limit of normal and increased by ≥25% or be reelevated to ≥200 U/mL over the previous value  - If reelevated to <2 times the upper limit of normal, the total creatine kinase must exceed the upper limit of normal by ≥50% and exceed the previous value by 2-fold or be reelevated to ≥200 U/mL  Reinfarction after percutaneous coronary intervention is defined as: creatine kinase - MB or (creatine kinase, if MB is not available) >3 times the upper limit of normal and ≥50% greater than the previous value and/or new Q waves (Minnesota Code) in 2 or more contiguous leads  Reinfarction after coronary artery bypass grafting surgery is defined as: creatine kinase - MB (or creatine kinase, if MB is not available) >5 times the upper limit of normal and ≥50% greater than the previous value and/or new Q waves (Minnesota Code) in 2 or more contiguous leads  **Aborted MI**:  Combination of chest pain and transient ECG changes (≥50% ST resolution) suggesting transmural ischemia, and creatine kinase/ creatine kinase-MB levels ≤2 times the upper limit of normal and/or troponin I/T levels corresponding to this creatine kinase/ creatine kinase-MB metric within 24 h after randomization  **Major bleeds**:  Severe bleed—a bleed that leads to a hemodynamic compromise requiring intervention (eg, blood or fluid replacement, inotropic support, ventricular assist device, surgical repair), or life-threatening or fatal bleeds.  Moderate bleed—bleeding requiring transfusion of blood but that does not lead to hemodynamic compromise requiring intervention. |
| Sinnaeve, 2014 | **Congestive heart failure**:  A positive diagnosis consists of at least one of the following conditions requiring treatment with diuretics:  - Pulmonary oedema/congestion on chest x-ray without suspicion of a non-cardiac cause;  - Rales >1/3 up from the lung base (Killip class 2 or higher);  - Pulmonary capillary wedge pressure >25 mmHg;  - Dyspnea with pO2 < 80 mmHg or O2 sat < 90 % (no supplemental O2) in the absence of known lung disease  **Cardiogenic shock**:  Defined as one of the following:  1. Systolic blood pressure < 90 mmHg for at least 30 min (or the need for supportive measures to maintain a systolic blood pressure of > 90 mmHg) in the presence of a heart rate of >60 beats/min in association with signs of end organ hypoperfusion (cold extremities, low urinary output < 30 ml/h and/or mental confusion);  2. A cardiac index < 2.2 l/min/m2 in the presence of pulmonary capillary wedge pressure of >15 mmHg.  **Reinfarction**:  In the first 18 hours after randomization reinfarction is defined as:  - recurrent signs and symptoms of ischemia at rest, accompanied by new or recurrent ST-segment elevations of ≥ 0.1 mV in at least two contiguous leads lasting ≥ 30 min.  After 18 hours reinfarction is defined as follows:  - new Q waves (by Minnesota Code Criteria) in two or more leads and/or enzyme/ biochemical evidence of reinfarction: re-elevation of creatine kinase-MB or troponin to above the upper limit of normal and increased by ≥ 50% over the previous value;  - if creatine kinase-MB or troponin is not available, the total creatine kinase will be evaluated: 5  - the total creatine kinase must either be re-elevated to ≥ 2 times the upper limit of normal and increased by ≥ 25 % or be re-elevated to ≥ 200 U/ml over the previous value;  - if re-elevated to < 2 times the upper limit of normal, the total creatine kinase must exceed the upper limit of normal by ≥ 50 % and exceed the previous value by two-fold or be re-elevated to ≥ 200 U/ml.  Reinfarction after percutaneous coronary intervention is defined as:  - creatine kinase-MB (or creatine kinase, if MB is not available) > 3 times the upper limit of normal and ≥ 50 % greater than the previous value and/or new Q waves (Minnesota Code) in two or more contiguous leads.  Reinfarction after coronary artery bypass grafting surgery surgery is defined as:  - creatine kinase -MB (or creatine kinase, if MB is not available) > 5 times the upper limit of normal and ≥50% greater than the previous value and/or new Q waves (Minnesota Code) in two or more contiguous leads.  **Bleeds**:  Major bleeds  Severe bleed: a bleed that leads to hemodynamic compromise requiring intervention (e.g. blood or fluid replacement, inotropic support, ventricular assist device, surgical repair) or life-threatening or fatal bleeds.  Moderate bleed: bleeding requiring transfusion of blood but which does not lead to hemodynamic compromise requiring intervention.  Minor bleeds  Mild bleed: bleeding neither requiring blood transfusion nor leading to hemodynamic compromise. |
| Pu, 2017 | **Death**:  Death will be classified as cardiovascular or non-cardiovascular. All cause deaths will be considered cardiac unless a definite noncardiac cause can be established. **Reinfarction**:  1.Reinfarction within 18 hours of onset of the index myocardial infarction: new ST elevation of ≥1 mm in at least 2 contiguous leads and recurrent cardiac ischemic symptoms ≥20 min at rest.  2.Reinfarction after 18 h of onset of the index myocardial infarction but before myocardial necrosis biomarkers have returned to normal: myocardial necrosis biomarker re-elevation (troponin) defined as an increase of ≥50% over a previous value that was decreasing, and at least one of the following: recurrent cardiac ischemic symptoms >20 min at rest, or one of the following ECG changes: new ST-segment elevation ≥1 mm in at least 2 contiguous leads, or development of new pathological Q waves on the ECG, or new left bundle branch block.  3.Reinfarction after myocardial necrosis biomarkers have returned to normal (excluding myocardial infarction in patients undergoing percutaneous coronary intervention in the previous 24 h): elevation of myocardial necrosis biomarkers typical of acute myocardial infarction, with at least one of the following: recurrent cardiac ischemic symptoms ≥20 min at rest, or development of new pathological Q waves on the ECG, or ECG changes indicative of ischemia, or pathological findings of an acute myocardial infarction.  4.Reinfarction within 24 h after percutaneous coronary intervention: Troponin ≥3 times the upper limit of normal and, if the pre-percutaneous coronary intervention troponin was > upper limits of normal, both an increase by ≥50% over the previous value, and documentation that troponin was decreasing prior to the suspected recurrent myocardial infarction (no symptoms are required), or development of new pathological Q waves on the ECG (no symptoms are required). **Heart failure:**  Patients presenting with at least one of the following conditions and requiring treatment with diuretics: 1) Pulmonary oedema/congestion on chest x-ray without suspicion of a non-cardiac cause; 2) Rales >1/3 up from the lung base; 3) Pulmonary capillary wedge pressure >25 mmHg; 4) Dyspnea with pO2 < 80 mmHg or O2 sat < 90 % (no supplemental O2) in the absence of known lung disease. **Stroke**:  Any stroke is defined as the presence of a new focal neurologic deficit thought to be vascular in origin, with signs or symptoms lasting more than 24 hours. It is strongly recommended (but not required) that an imaging procedure such as a computerized tomography or magnetic resonance imaging be performed. **Bleeding**:  Incidence of bleeding events will be classified by the GUSTO severity criteria15. GUSTO criteria for classifying the severity of bleeding complications: Severe or life-threatening bleeding: Intracranial bleeding or bleeding that causes substantial hemodynamic compromise requiring treatment; Moderate bleeding: Bleeding which needs blood transfusion; Minor bleeding: Other bleeding, neither requiring transfusion nor causing hemodynamic compromise. |
