# Supplementary material for: Reperfusion Strategy of ST-Elevation Myocardial Infarction: A Meta-Analysis of Primary Percutaneous Coronary Intervention and Pharmaco-Invasive Therapy
Source: Front Cardiovasc Med. 2022 Mar 17;9:813325. doi: 10.3389/fcvm.2022.813325 (PMC8970601; doi:10.3389/fcvm.2022.813325)
Supplement: Supplementary Table 6 — Outcome definitions of included observational studies. [file Table_6.DOCX]

Table S6. Outcome definitions of included observational studies.

| Study | Outcome definitions |
| --- | --- |
| Victor, 2014 | **Myocardial Infarction:** Detection of rise and/or fall of cardiac biomarker values (preferably troponin) with at least one value above the 99th percentile of the upper reference limit and with at least one of the following: -Symptoms of ischemia;  -New or presumably new significant ST-T changes or new left bundle branch block; -Development of pathological Q waves in the ECG; -Imaging evidence of new loss of viable myocardium, or new regional wall motion abnormality; -Identification of an intracoronary thrombus by angiography or autopsy.  **TIMI bleeding criteria:** (Non-coronary artery bypass grafting related bleeding) Major -Any intracranial bleeding (excluding microhemorrhages <10 mm evident only on gradient-echo Magnetic Resonance Imaging) -Clinically overt signs of hemorrhage associated with a drop in hemoglobin of ≥5 g/dL -Fatal bleeding (bleeding that directly results in death within 7 days) Minor -Clinically overt (including imaging), resulting in hemoglobin drop of 3 to <5 g/dL. Requiring medical attention -Any overt sign of hemorrhage that meets one of the following criteria and does not meet criteria for a major or minor bleeding event, as defined above. -Requiring intervention (medical practitioner-guided medical or surgical treatment to stop or treat bleeding, including temporarily or permanently discontinuing or changing the dose of a medication or study drug). -Leading to or prolonging hospitalization -Prompting evaluation (leading to an unscheduled visit to a healthcare professional and diagnostic testing, either laboratory or imaging) Minimal -Any overt bleeding event that does not meet the criteria above  **Cardiogenic shock:** Systolic blood pressure <90mm Hg for at least 30 min (or the need for supportive measures to maintain a systolic blood pressure of >90mmHg) in the presence of a heart rate of >60beat/min in association with signs of end-organ hypoperfusion (cold extremities, low urinary output <30mL/h and/or mental confusion) A cardiac index <2.21L/(min m2) in the presence of a pulmonary capillary wedge pressure of >15mmHg. **Killip Class:** Class I: no heart failure Class II: crackles audible half way up the chest Class III: crackles heard in all the lung fields Class IV: cardiogenic shock **Reinfarction:** Re-infarction was defined as having at least 2 of the following 4 criteria: (1) recurrent ischemic symptoms lasting >15 minutes after resolution of symptoms of the index myocardial infarction, (2) occurrence of new ST-T wave changes or new Q waves, (3) a second elevation in cardiac enzymes to over the normal upper limit (or by a further 20% if already over the normal upper limit), and (4) angiographic re occlusion of a documented previously patent infarct-related artery.  **Successful and Failed Thrombolysis:** Successful reperfusion is defined as resolution of chest pain, presence of reperfusion arrhythmias, and ST segment resolution >50% in the lead with maximum ST elevation in pre-Thrombolytic ECG. Failed thrombolysis is defined as persisting or worsening chest pain or <50% resolution of ST segment elevation after 90 minutes of thrombolysis in a single lead showing maximum ST segment elevation at presentation. |
| Danchin, 2008 | **Recurrent myocardial infarction** was defined as recurrent symptoms with a new rise in cardiac markers. Isolated troponin reelevation after PCI was not considered recurrent myocardial infarction in the absence of recurrent symptoms.  **Major bleeding** was defined as any fatal or life-threatening bleeding or bleeding associated with a 15% decrease in hematocrit, a 5-g/dL fall in hemoglobin, or intracranial bleeding. |
| Bainey, 2019 | International Classification of Diseases (ICD)-10 codes used for defining clinical events between discharge and one year:  **Cardiogenic shock**: R57.0  **Congestive heart failure**: I50.x  **Intracranial hemorrhage**: I60.x, I61.x, I62.x  **Major bleeding**: K25.(0,2,4,6), K26.(0,2,4,6), K27.(0,2,4,6), K28.(0,2,4,6), K92.(0,1,2), K29.x, K31.80, K31.88, K55.8, K55.20, K62.5, K63.80, K63.88, K91.80, I60.x, I61.x, I62.x, S06.(4,5,6,7), R04.x, I85.0, I98.2  **Myocardial infarction**: I21.x, I22.x |
| Araiza-Garaygordobil, 2021 | The primary composite end point included the occurrence of cardiovascular death, cardiogenic shock, recurrent myocardial infarction (MI), or congestive heart failure at 30 days of follow-up; the key safety end points included the proportion of patients with major bleeding (Bleeding Academic Research Consortium [BARC] score 3-5) at 30-day follow-up and the proportion of patients with intracranial hemorrhage.  Outcomes were on the basis of international standardized definitions for end points in clinical trials, as stated in 2017 Cardiovascular and Stroke Endpoint Definitions for Clinical Trials (DOI: 10.1016/j.jacc.2017.12.048). |
| Zubaid, 2020 | **Congestive heart failure** was defined by the development of symptoms, signs or radiological evidence of pulmonary edema/congestion requiring diuretic therapy.  **Reinfarction** was defined as recurrent signs and symptoms of ischemia at rest, accompanied by new or recurrent ST-segment elevations of ≥0.1 mV in at least two contiguous leads lasting ≥30 minutes.  **Stroke** was defined as rapidly developing clinical signs of focal (or global) disturbance of cerebral function, with symptoms lasting 24 hours or longer or leading to death, with no apparent cause other than of vascular origin.  **Bleeding**: The Bleeding Academic Research Consortium (BARC) criteria was used to classify bleeding types. Major bleeding was defined as BARC type 2 or higher, that is any overt sign of hemorrhage that is actionable and requires diagnostic studies, hospitalization or treatment by health care professional. |
| AG, 2018 | Not clarified. |
| Rashid, 2016 | **Stroke** was defined as the presence of new neurological deficits lasting for >24 h with evidence of ischemia or hemorrhage demonstrated by computed tomography or magnetic resonance imaging.  **Reinfarction** was defined as the recurrence of cardiac ischemic symptoms occurring at rest and lasting for >30 min in combination with reelevation of ST-segment on an electrocardiogram and of cardiac enzymes (twice the upper limit of normal) or angiographic evidence of reocclusion of the infarct-related artery.  **Bleeding**: Major and minor bleeding were defined according to the TIMI classification. |
| Chava, 2014 | **Mortality** was defined as all-cause death during the index hospitalization.  **Bleeding** complications were categorized on the basis of HORIZONS and TIMI major bleeding scales.  **Acute stent thrombosis** was defined according to modified Academic Research Consortium criteria. |
| Bodı ́, 2011 | Not clarified. |
| Auffret, 2019 | The primary end point of the study was in-hospital major adverse cardiovascular events (MACE), defined as the composite of all-cause mortality, nonfatal myocardial infarction, stroke and definite stent thrombosis (ST) according to the Academic Research Consortium definition. Secondary end points included all-cause death, major bleeding recorded since the inclusion of the Bleeding Academic Research Consortium (BARC) definition in the ORBI database in 2011 and defined as BARC 3 or 5 bleeding, net adverse clinical events defined as the composite of MACE and major bleeding, and calculated in patients with data regarding inhospital BARC-defined bleeding events, and development of in-hospital Killip class III or IV heart failure. |
| Sim, 2016 | **Recurrent MI** was defined as the recurrence of symptoms or the presence of ECG changes in association with a rise in cardiac markers above the upper limit of normal.  **Target-vessel revascularization** was defined as a repeat percutaneous coronary intervention of any segment within the entire major coronary vessel proximal and distal to a target lesion, including the target lesion itself.  **Major bleeding** was defined as type 3 or type 5 of the Bleeding Academic Research Consortium. |
| Jarle Jortveit, 2021 | **Major Bleeding:** (i) fatal bleeding, (ii) bleeding in a critical area or organ, such as intracranial, intraspinal, retroperitoneal, intraocular, intra-articular or pericardial, or intramuscular with compartment syndrome, and/or (iii) bleeding with haemoglobin drop >5 g/dL or ≥2 blood transfusions. |
